# Efficacy of Tree Trunk Coating Materials in the Control of the Apple Clearwing, *Synanthedon myopaeformis*

**DOI:** 10.1673/031.010.6301

**Published:** 2010-06-14

**Authors:** Fedai Erler

**Affiliations:** Akdeniz University, Faculty of Agriculture, Department of Plant Protection, 07070 Antalya, Turkey

**Keywords:** Apple clearwing, used motor oil, cotton seed oil, lime, trunk treatment, apple tree

## Abstract

The efficacy of trunk treatment with three materials, cotton seed oil, lime and used motor oil, were evaluated for the control of apple clearwing, *Synanthedon myopaeformis* (Borkhausen) (Lepidoptera: Sesiidae) in an apple orchard during two successive years (2004 and 2005). The weekly total number of adult catches and exuviae was recorded each year. No treatments caused significant reductions in mean numbers of adults caught in bait traps or the exuviae protruding from the barks of tree trunks and thick branches in the first year of the study whereas all of them differed significantly from each other or from water-treated control in the second year (*P* < 0.05). A comparison of the mean numbers of adult catches and exuviae in both years revealed significant differences between the used motor oil and cotton seed oil treatments (*P* < 0.05). The lime treatments in both years significantly differed in terms of adult catches, but not exuviae (*P*<0.05). In the second year, compared with those in water-treated control plots, the mean number of adult catches and exuviae decreased by 81.3% and 88.3% in the used motor oil-treated plots, and by 70.8% and 83.3% in the cotton seed oil-treated plots, respectively. Although population reductions in the lime treatment were significant in the second year, the effect was at a much reduced level in comparison to the two oil treatments. The overall results suggest that used motor oil and cotton seed oil may have potential for the control of apple clearwing.

## Introduction

The apple clearwing, *Synanthedon myopaeformis* (Borkhausen) (Lepidoptera: Sesiidae), is a xylophagous species that attacks pome and stone fruit trees (Mustafa and Sharaf 1994; Canadian Food Inspection Agency 2006). The larval form of this insect lives under the bark of fruit trees, especially apple (*Malus*), but sometimes pear (*Pyrus*), almond (*Prunus amygdalus* Batsch) and a few other closely related plant species (Bartsch 2004). The larvae located under the bark of tree trunk and thick branches bore deep sub-cortical galleries 20 to 25 mm long and cut into the phloem (Dickler 1976; Iren and Bulut 1981). The control of this pest is difficult because the adults have a long emergence period and the larvae develop inside the trunk and thick branches. Failure to prevent injury can lead to reduced tree vigor and yield (Iren et al. 1984; Kovancı 1986).

Until the 1980's *S. myopaeformis* has been regarded as a secondary pest of apple trees weakened by other factors, but in the last decade it has become a serious pest of apple trees in Antalya, in southwestern Turkey, and other parts of the country(Zeki et al. 1998). This can be attributed to changes in apple production technology and pest control strategies (Ateyyat 2006). Chemical treatments that were locally applied only onto trunk and thick branches, or generally applied onto entire tree using broad spectrum insecticides previously provided control of apple clearwing (Ulu et al. 1983; Iren et al. 1984; Maçan et al. 1987; Kılıç et al. 1988; Ateyyat 2005). According to several previous studies (Dickler 1976; Frankenhuyzen and Jansen 1978; Ateyyat and Al-Antary 2006), trunk treatment with various materials (carboxyl methyl cellulose, endosulfan, ethyl-parathion, mineral oil, polyvinyl acetate etc.) against this pest reduced *S. myopaeformis* populations below the economic threshold that is, according to the Technical Bulletin of Turkish Ministry of Agriculture (Zeki et al. 1998), 8–10 larvae per tree during MarchOctober. Also, Maçan et al. (1987) reported that only trunk and thick branch spraying with several broad spectrum insecticides (including chlorpyriphos ethyl and azinphos methyl) effectively controlled larvae of *S. myopaeformis.* However, many of these broad spectrum insecticides are less frequently recommended in integrated pest management (IPM) programs, due to their negative effects on natural enemies (Trematerra 1993; Balázs et al. 1996).

The need for new materials to reduce *S. myopaeformis* populations prompted field studies to determine the potential of several materials as control agents and to determine whether trunk treatment alone as a control procedure could be enough to reduce the *S. myopaeformis* populations.

## Materials and Methods

### Test materials

The test materials used were cotton seed oil [Antbirlik Corp. Ltd., Antalya, Turkey; density = 0.94 g/ml, containing linoleic acid (49–58%), palmitic acid (22–26%), oleic acid (15–20%)), and a 10% mixture of arachidic acid, behenic acid and lignoceric acid], lime [Taspinar Ltd., Antalya; hydrated, 481 kg/cu.m., the primary active component is calcium carbonate, additional components are: calcium oxide, magnesium oxide and magnesium carbonate] and used motor oil [the Castrol-Turkish distributor, Antalya; weight (w) = 20W-50], The choice of cotton seed oil and used motor oil was based upon comprehensive data on the use of oils as insecticides and acaricides (Chapman 1967; Butler et al. 1988; Willett and Westigard 1988; Butler and Henneberry 1990; Erler 2004). Lime, is traditionally used in Turkey to protect park and garden trees from insects and fungal pathogens by whitewashing their trunks.

### Experimental site and design

Trials were carried out in a 0.37 ha orchard comprised of 98 ‘Starking’ and eight ‘Golden Delicious’ trees (the latter were excluded from the study) in Korkuteli, located at an altitude of ∼1000 m, near Antalya during the 2004 and 2005 growing seasons. The orchard was abandoned and free from pesticide sprays since 1998. The trees were 19–20 years old and heavily infested by *S. myopaeformis.* The trees were grouped for treatments in rows and treatments were applied in a completely randomized block design in three replications, with a water-treated control plot in each replicate; each plot consisted of eight trees.

### Applications

Four applications were made during each growing season. The first was made at the start of adult emergence when first moths were detected in the bait traps during the first half of May. Detection of adult emergence and observation of the flight of adults was based on bait traps. Other applications were made at one month intervals, following the first. All test materials were applied onto the surface of tree trunks and thick branches by using a largehand brush. The entire trunk area and the first 50 cm of primer thick branches, ≥16 cm in diameter, were treated with the test materials. The oils were applied directly (without emulsification). The lime was applied as an aqueous solution (30% lime in water).

### Sampling and data collection

In each year, treatments were evaluated by counts of adults trapped and pupal skin. Adult trapping was carried out in both years by placing bait traps from the beginning of April to the end of September. Cylindrical plastic containers (18 × 18 cm; diameter × height) modified for this purpose were used as bait traps. Each trap contained 0.5 1 of bait consisting of 80% water, 20% grape molasses and 2–3 g yeast. Six traps per treatment (2 per plot) were positioned at a height of about 1.6 m in apple trees randomly selected from the center areas of each plot. All traps were checked and cleaned on a weekly interval until late September. The numbers of exuviae protruding from the barks of tree trunks and thick branches were also determined weekly. On each sample date, the entire circumference of the trunk and the first 50 cm of treated main branches were inspected for exuviae on each of 6 trees per treatment (two per plot). The trees were selected at random in each plot at the beginning of the study and marked with colored plastic strips. To eliminate recounting the same skins at the next samplings, the exuviae protruding from the bark were removed by pulling out with a fine forceps after being counted.

### Statistical analysis

Data obtained from the weekly samplings were analyzed by ANOVA (SAS Institute 2001) and converted to yearly mean number of adults caught per trap or of exuviae per tree for each treatment. The Tukey's test was used to separate differences in means among the treatments. Yearly percent decreases in adult catches and exuviae for each treatment were also calculated by using a formula [decrease (%) = 100 × (A–B)/A, where A is yearly mean number of adult catches per trap or of exuviae per tree in the control; and B is yearly mean number of adult catches per trap or of exuviae per tree in the treatment, as defined by Rice and Coats (1994)]. In addition, the weekly mean numbers of adult catches and exuviae are presented in graphic form.

## Results and Discussion

**Figure 1.**
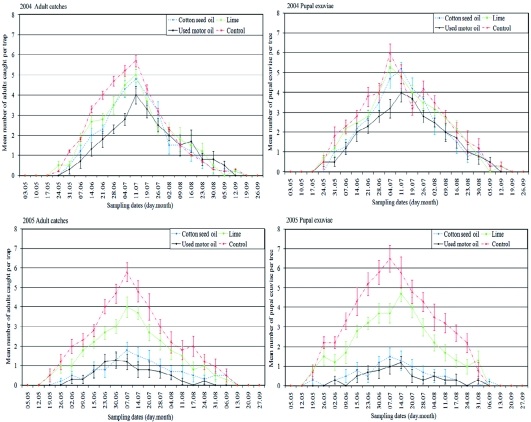
Weekly mean numbers of apple clearwing adults caught in bait traps and exuviae protruding from trees during weekly examinations from early May to late September in 2004 and 2005 growing seasons. Means (with Standard errors) from 6 bait traps or 6 trees, adults and exuviae, respectively. High quality figures are available online.

The weekly mean numbers of adults caught in bait traps and of exuviae protruding from the barks of tree trunks and thick branches in both growing seasons are presented in Figure 1. Adult flight began at the beginning of May, reached a maximum at the beginning or in the middle of July, and ended at the end of September. The weekly total numbers in treated plots were less than those in watertreated control plots in both years. However, numbers varied considerably from year to year, and more substantial treatment effects were observed in the second year of the study (Figure 1).

Test materials varied in their impact on *S. myopaeformis* (Table 1). Used motor oil, cotton seed oil and lime treatments in the first year of the study did not differ significantly from each other or from water-treated control in terms of the mean numbers of adults caught and exuviae. This suggests that the treatment had minimal impact on developing larvae.

In contrast, there were significant differences between the test materials and water-treated control in the second year (*P*<0.05). When compared on the basis of mean numbers for the same materials over two different years, the used motor oil and cotton seed oil treatments were significantly different from each other (*P*<0.05). There were no significant differences between the lime treatments in both years (*P*<0.05). The used motor oil and cotton seed oil treatments caused 32.8% and 20.7%) reductions in mean number of adults caught in the first year, 81.3% and 70.8% in the second year, respectively. Reductions in mean numbers of exuviae were at approximately the same rates (Table 1). The lime treatment caused significant reduction only in mean number of adults caught in the second year of the study. However, this decrease was significantly less than those in oil treatments and was not statistically different from the water only control.

There was no difference in appearance of treated trees as an indication of phytotoxicity on plant tissue during the study compared with water-treated controls. However, it must be kept in mind that the oil treatments affect stomatal openings and can cause damage by reduced plant transpiration in the course of time. In addition, more attention must be paid to the use of used motor oil due to its different structure from oils used as insecticides. It is suggested that it should not be used before detailed investigations on its phytotoxicity are made and it takes an approval for use from Turkish Ministry of Agriculture.

**Table 1.**
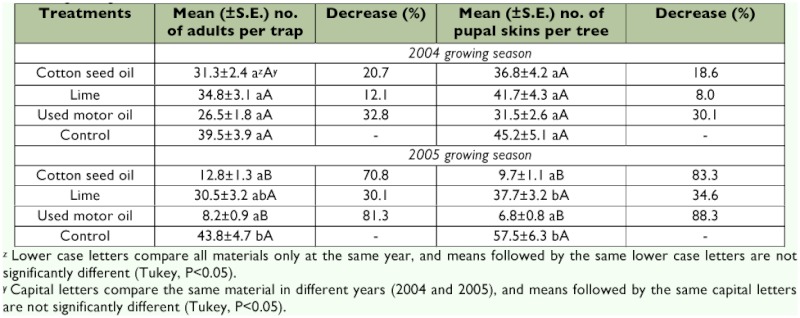
Yearly mean numbers of adult catches and pupal skins, and their percent decreases in the treatment plots in 2004 and 2005 growing seasons

The results obtained from the study revealed *S. myopaeformis* flight activity from early May to late September and adult flight reached a maximum at the beginning or in the middle of July. This coincides with the work of Al-Antary and Ateyyat (2006), except for flight peak of the insect. Al-Antary and Ateyyat (2006) reported that adults of *S. myopaeformis* had two flight peaks in the Ash-Shoubak apple-growing region of southern Jordan; the first one was between 11th and 18th June and the second was in mid-July.

The results also revealed that tree trunk treatment with oil substances significantly reduced the insect populations compared with water-treated control. The fact that the number of trapped adults and pupal cases protruding motor oil and cotton seed oil treatments in the second year may be attributed to the low egglaying activity of the females in oily surfaces in the first year of the study. Taking into consideration results from previous studies (Butler and Hennebery 1991; Fenigstein et al. 2001; Erler 2004; Erler et al. 2007), it can be surmised that the oils likely act as settling and oviposition deterrents. Also, Dickler (1976) reported that apple clearwing populations were enormously reduced in two years by trunk treatments with the combination of ethyl parathion and mineral oil, and that the applications were made at one month intervals, from the beginning of April to the end of August. In a previous study by Ateyyat and Al-Antary (2006), various treatments including (1) using a flexible wire to mechanically kill the larvae, (2) painting the trunk of trees with a mixture of water, copper sulfate, petroleum oil, and Durusban® (chlorpyriphos), (3) mounding soil to cover the graft union area and (4) a cloth veil wrapped around the main tree trunk from its base up to a height of 80 cm were evaluated for the control of *S. myopaeformis* and the insecticidal paint treatment was found to cause the greatest population reduction.

Irrespective of origin, oils are generally considered physical suffocants which interfere with respiration in insects and mites (Chapman 1967; Butler et al. 1988; Willett and Westigard 1988; Butler and Henneberry 1990, 1991). In the present study, decreases in mean numbers of adults caught and exuviae in used motor oil and cotton seed oil plots in the first year of the study were substantially less than those in the second year. Taking into consideration the economic threshold value, the first year results of the oil treatments were higher than the economic threshold whereas the second year results were under the economic threshold (Zeki et al. 1998). This indicates that the larvae developing inside the tree trunks and thick branches were affected by the oil treatments to a limited extent.

In conclusion, the trunk treatment with used motor oil and cotton seed oil as a control procedure caused significant decreases in the population density of *S. myopaeformis,* showing that it may be a useful method to control this pest. Moreover, the relatively low cost of these oils compared to most commercial insecticides, low or no mammalian toxicity and reduced potential for development of arthropod resistance make them attractive candidates for use in the control of this pest.
